# Dermatofibrosarcoma Protuberans Arising in a Decorative Tattoo

**DOI:** 10.1080/13577140500094289

**Published:** 2005

**Authors:** Paul A. Baker, Geraldine J. O'Dowd, Irfan U. Khan

**Affiliations:** 1 Canniesburn Plastic Surgery Unit Glasgow Royal Infirmary Glasgow G4 0SF Scotland; 2 Department of Pathology Monklands District General Hospital Monkscourt Avenue ML6 0JS Scotland; 3 Flat 3/1 40 Craigpark Drive Dennistoun Glasgow G31 2NP Scotland

## Abstract

Dermatofibrosarcoma protuberans (DFSP) is an uncommon, locally aggressive cutaneous tumour of intermediate grade malignancy. A number of reports have linked local trauma of varying aetiology with the later development of DFSP. In addition, a variety of skin disorders and, in rare cases, cutaneous tumours, have been described in association with decorative tattoos. This is often associated with delayed diagnosis. We report the first case of DFSP arising in a tattoo and discuss the available evidence for a causative link between DFSP and local trauma of this nature.


*Sarcoma*, March/June 2005; 9(1/2): 37–41

Taylor & Francis

Taylor & Francis Group

CASE REPORT

### Dermatofibrosarcoma protuberans arising in a decorative tattoo

PAUL A. BAKER[1], GERALDINE J. O'DOWD[2], & IRFAN U. KHAN[1]

[1]*Canniesburn Plastic Surgery Unit, Glasgow Royal Infirmary, Glasgow, Scotland G4 0SF, UK, and*
[2]*Department of Pathology, Monklands District General Hospital, Monkscourt Avenue, Scotland ML6 0JS, UK*

**Abstract**
Dermatofibrosarcoma protuberans (DFSP) is an uncommon, locally aggressive cutaneous tumour of intermediate grade malignancy. A number of reports have linked local trauma of varying aetiology with the later development of DFSP. In addition, a variety of skin disorders and, in rare cases, cutaneous tumours, have been described in association with decorative tattoos. This is often associated with delayed diagnosis. We report the first case of DFSP arising in a tattoo and discuss the available evidence for a causative link between DFSP and local trauma of this nature.

**Keywords:** *Dermatofibrosarcoma protuberans, tattoo*

### Introduction

Dermatofibrosarcoma protuberans (DFSP) is a rare neoplasm of intermediate grade malignant potential. It is characterised by latency in its initial detection, slow infiltrative growth, and local recurrence if inadequately excised. The tumour is locally aggressive and rarely metastasises [1, 12, 13].

A number of authors have reported cases of DFSP arising at sites of antecedent local trauma of varying aetiology and have suggested a causal relationship. Those cases reported to date include the development of DFSP at sites of surgical scars, burn scars, and at sites of prior immunisation and therapeutic irradiation [2–7, 25–30].

In addition, a wide range of local and systemic complications have been described in association with decorative tattooing. These include allergic reactions, infections and cutaneous disorders such as psoriasis and seborrhoeic keratosis. More rarely, however, cutaneous neoplasms such as basal cell carcinoma, squamous cell carcinoma, and malignant melanoma have also been described in association with tattoos [9–11, 32–34].

This is the first report, to our knowledge, of DFSP occurring in a decorative tattoo.

### Case report

A 35-year-old man with no significant past medical history was referred by his general practitioner to the local general surgical service for assessment of a lesion arising in a tattoo. The tattoo was situated on the dorsum of his left forearm and had been present for approximately 8 years. Over the past 7 years, however, the patient had become aware of a slowly enlarging mass within the pigmented skin. The patient had previously attended his GP one year earlier with this problem and had been managed with reassurance.

On examination, he was found to have a non-tender, firm mass that was approximately 1 cm in diameter. The mass was mobile and did not appear to be attached to underlying tissues. There was no clinical evidence of associated regional lymphadenopathy. The lesion was thought to be a simple epidermoid cyst and local excision was performed with minimal margins.

The specimen, a ragged fragment of yellow-brown fibrous tissue (12 mm diameter), underwent histological assessment. This revealed prominent black, granular pigment within the superficial dermis, consistent with the history of tattooing at this site (Figure 1). The epidermis appeared within normal

Correspondence: Mr Paul A. Baker, Flat 3/1, 40 Craigpark Drive, Dennistoun, Glasgow, Scotland G31 2NP, UK. Tel: 44 141 211 4000. Fax: 44 141 211 5652. E-mail: paul_andrew_baker@hotmail.com

ISSN 1357–714X print/ISSN 1369–1643 online © 2005 Taylor & Francis Group Ltd
DOI: 10.1080/13577140500094289

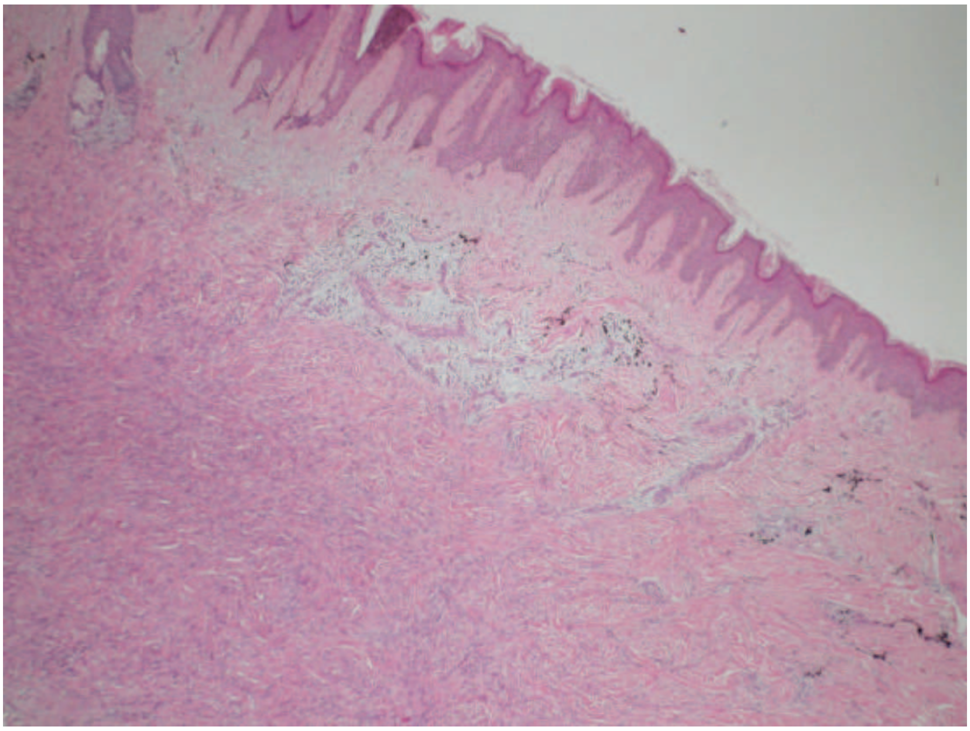

Figure 1. Black, granular tattoo pigment within superficial dermis. Note underlying spindle cell tumour (haematoxylin & eosin, original magnification, ×4).

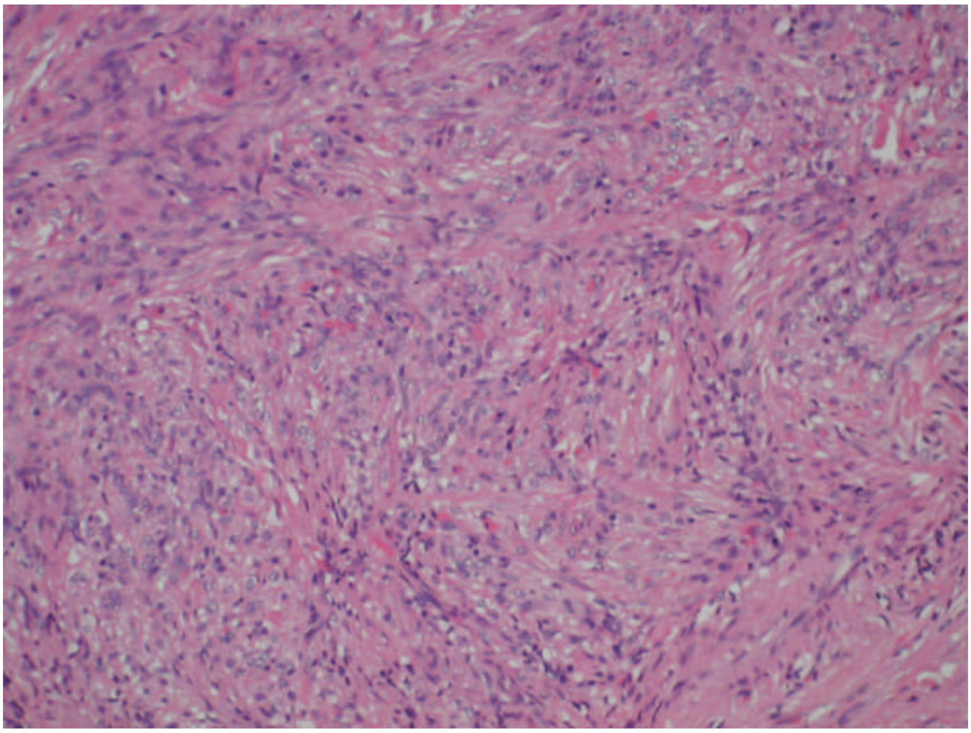

Figure 2. Moderately cellular tumour composed of bland, elongated cells with characteristic storiform arrangement (haematoxylin & eosin, original magnification, ×20).

limits. However, a poorly circumscribed tumour was present within the dermis that infiltrated into the superficial subcutaneous connective tissue. It was composed of slender spindle cells arranged focally in a storiform pattern (Figure 2), the hallmark of DFSP. These cells had features of intermediate grade malignancy. The lesion extended to the surgical margins. Immunohistochemical staining was strongly positive for vimentin and CD34 (Figure 3), supporting the histological diagnosis of DFSP.

As the lesion appeared to be incompletely excised, the patient was referred to the regional plastic

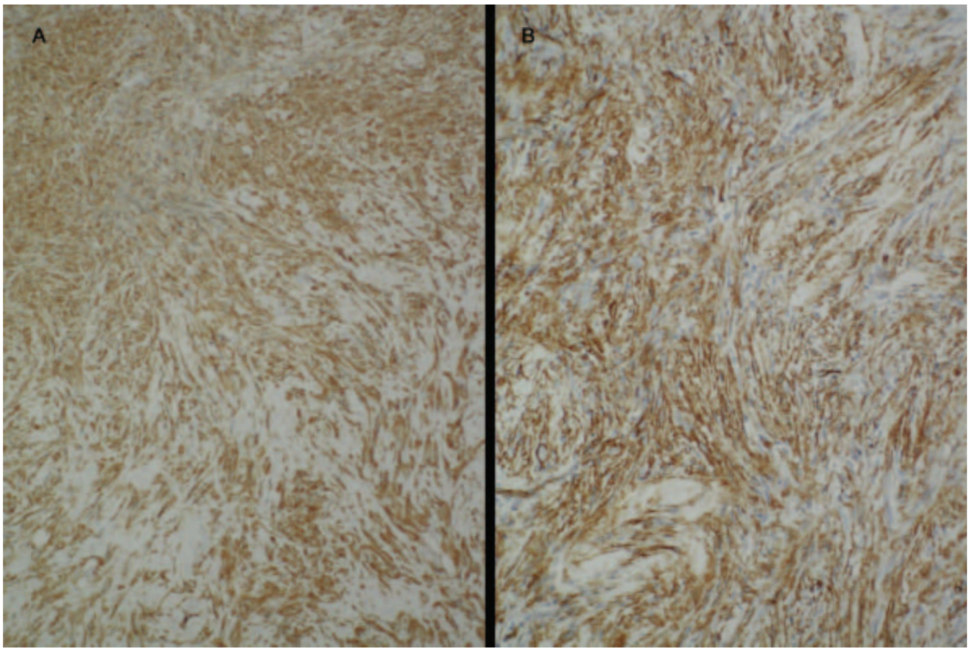

Figure 3. Immunohistochemical staining for vimentin (A) and CD34 (B) is strongly positive, consistent with the diagnosis of DFSP (original magnifications, ×20).

surgery service. A wider local excision was performed with a 4-cm margin and the defect covered with a split skin graft. Pathological examination of this specimen did not reveal any evidence of residual disease.

On follow-up, the patient continues to make a good recovery, with no evidence of recurrent or metastatic disease at 2 years.

### Discussion

### *Aetiology*

There have been several theories on the aetiology of DFSP [8]; however, the cause of malignant transformation is as yet unknown [12]. There does not appear to be a hereditary or familial predisposition and the precise role of recently reported cytogenetic aberrations has yet to be determined [12].

A number of previous reports have suggested a causal relationship between antecedent local trauma of varying aetiology and the subsequent development of DFSP. These include the development of DFSP at sites of trauma ranging from 'vaccination to bayonet wound' [2], such as surgical scars [25–27], burn scars [28,29], sites of prior immunisation [4–7] and therapeutic irradiation [12,30]. In addition, increased frequency of trauma to the hands and feet is thought to account for the higher incidence of acrally occurring DFSP in children and adolescents compared to adults [12]. Trauma preceded tumour development by periods ranging from 2 months to 20 years, although shorter periods tended to predominate [2]. Furthermore, a clinical history of previous local trauma was obtained in up to 10–20% of all cases of DFSP by some authors [2, 8, 12, 26].

It has been suggested that such connective tissue tumours occur much less frequently than epidermal malignancies because the deeper tissue is subjected to less trauma and undergoes less tissue regeneration than the superficial, more vulnerable, epidermis [28].

It is well known that scars are a predisposing factor in the development of cutaneous malignancy and there have been several previous reports of DFSP occurring in scars of varying aetiology [25–29]. The development of malignancy in vaccination scars is well recognised and, specifically, several previous case reports have documented the occurrence of DFSP in this setting [2, 4–7]. Local trauma, wound healing, scarring, infection and also antigenic stimulation or oncogene transduction by the inoculated infectious agents have been implicated in the pathogenesis of malignancy in these circumstances [6]. However, it is impossible to quantify the potential contribution of each of these factors in isolation in a single case.

In this case there was a history of focussed local trauma 1 year prior to the development of this tumour, which is suggestive of a causal relationship. Interestingly, some authors have shown that more rapid growth of the tumour appears to be associated with an episode of trauma [2]. This unique method of local trauma is previously undescribed in the debate on its role in the pathogenesis of this rare tumour. Tattooing does, however, cause local trauma in a mode similar to that resulting from injections and multiple immunisations, a setting where DFSP has previously been reported [4–7, 28]. It is also possible that persistent inflammation and wound healing associated with the potentially toxic inoculated tattoo pigment, as with inoculated infectious agents, may have contributed to the subsequent development of DFSP in this case.

*Clinical aspects*

This case illustrates that DFSP is easily dismissed by both patient and physician as a benign skin condition not requiring biopsy. Furthermore, DFSP rarely presents as a firm cutaneous nodule as in this case and is infrequently located on the forearm [2]. The relative paucity of symptoms, non-specific clinical appearances and indolent behaviour of DFSP often tends to delay presentation and has led to a variety of clinical misdiagnoses [8, 12, 20, 26]. In addition, the presence of tattoo pigments in this case led to a further delay in the presentation, clinical diagnosis and treatment. This is a cause for concern and serves to remind us of previously reported cases of tattoos masking important diagnoses such as malignant melanoma [11, 34].

Decorative tattooing has been associated with a wide range of local and systemic complications. These include allergic reactions and infections. Many primary cutaneous disorders such as psoriasis, lichen planus and discoid lupus erythematosus have been reported to localise in tattoos [33]. Other lesions which have been described in association with tattoos include seborrhoeic keratosis [10] and, more rarely, cutaneous tumours such as basal cell carcinoma [9, 35], squamous cell carcinoma [36], reticulohistiocytoma [9] and malignant melanoma [11, 34]. This is the first report, to our knowledge, of DFSP occurring in a decorative tattoo.

### Conclusions

A growing body of circumstantial evidence links DFSP with previous local trauma. However, the nature and severity of the trauma and the interval between it and the onset of the tumour varies greatly. Evidence for a clear causal relationship is lacking due to limitations imposed by the relative rarity of these tumours and by the nature of retrospective analysis. The extent of this association and its underlying mechanisms therefore remains uncertain. The question therefore remains of trauma acting as a trigger in predisposed individuals or simply attracting attention to a previously existing mass.

This is the first report of DFSP arising in this setting. The focus of this article is to report that a sarcoma such as DFSP has developed at the site of a tattoo after 1 year. When viewed in this context it seems reasonable to conclude that the tattoo has had some contribution to the process of malignant transformation. In documenting this occurrence we contribute to the debate and to the body of circumstantial evidence linking various types of local trauma to the pathogenesis of this rare tumour. We speculate that DFSP may have occurred as a rare delayed complication at the site of a tattoo. This mode of local trauma is similar to that sustained at sites of injection and immunisation where the tumour has previously been described [4–7, 28]. We also add to the list of various cutaneous tumours documented as occurring at the site of decorative tattooing by contributing a case previously undescribed. Furthermore, we use this case to remind doctors to remain vigilant to the possibility of important diagnoses masked by tattoo pigments and draw attention to the diverse array of pathologies that have been described and masked in this setting.

### Acknowledgements

We would like to thank Mr S.B. Watson FRCS (Plast), Consultant Plastic and Hand Surgeon, Canniesburn Plastic Surgery Unit, Glasgow Royal Infirmary, UK, for allowing us to report on his patient and for reviewing the manuscript.

### References

[1] Weiss SW, Goldblum JR. Fibrohistiocytic tumours of intermediate malignancy. In Weiss SW, Goldblum JR, editors. Enzinger and Weiss's soft tissue tumours, 4th ed. St. Louis, MO: Mosby; 2001.

[2] Taylor HB, Helwig EB. Dermatofibrosarcoma protuberans: A study of 115 cases. Cancer 1962;15:717.

[3] Holm J. Dermatofibrosarcoma protuberans: report of a case with review of the literature. Acta Chirurgica Scandinavica 1968;134:303.

[4] McLelland J, Chu T. Dermatofibrosarcoma protuberans arising in a BCG vaccination scar. Archives of Dermatology 1988;124:496.

[5] Morman MR, Lin RY, Petrozzi JW. Dermatofibrosarcoma protuberans arising in a site of multiple immunisations. Archives of Dermatology 1979;115:1453.

[6] Green JJ, Heymann WR. Dermatofibrosarcoma protuberans occurring in a smallpox vaccination scar. Journal of the American Academy of Dermatology 2003;48:S54.

[7] Elgart GW, Hanley A, Busso M, Spencer JM. Bednar tumour (pigmented dermatofibrosarcoma protuberans) occurring in a site of prior immunisation: Immunochemical findings and therapy. Journal of the American Academy of Dermatology. 1999;40:315.

[8] Pack GT, Tabah EJ. Dermatofibrosarcoma protuberans. Archives of Surgery 1951;62:391.

[9] Wiener DA, Scher RK. Basal cell carcinoma arising in a tattoo. Cutis; Cutaneous Medicine for the Practitioner 1987; 39(2):125.

[10] Nicolle E, Bessis D, Guilhou JJ. Seborrhoeic keratosis erupting in a tattoo. Annales de Dermatologie et de Vénéréologie 1998;125(4):261.

[11] Stinco G, De Francesco V, Frattasio A, Quinkenstein E, Patrone P. Malignant melanoma in a tattoo. Dermatology 2003;206(4):345–346.

[12] Gloster HM Jr. Dermatofibrosarcoma protuberans. Journal of the American Academy of Dermatology 1996; 35:355–374.

[13] Rutgers EJ, Kroon BR, Albus-Lutter CE, et al. Dermatofibrosarcoma protuberans: treatment and prognosis. European Journal of Surgical Oncology: The Journal of the

European Society of Surgical Oncology and the British Association of Surgical Oncology 1992;18:241–248.
[14] Laskin WB. Dermatofibrosarcoma protuberans. CA: A Cancer Journal for Clinicians 1992;42(2):116–125.
[15] Roswell AR, Poole MD, Godfrey AM. Dermatofibrosarcoma protuberans: The problems of surgical management. British Journal of Plastic Surgery 1986;39:262–264.
[16] Sondak VK, Cimmino VM, Low LM, et al. Dermatofibrosarcoma protuberans: What is the best surgical approach? Surgical Oncology 1999;8:183–189.
[17] Bendix-Hansen K, Myhre-Jensen D, Haae S. Dermatofibrosarcoma protuberans: a clinico-pathological study of nineteen cases and review of world literature. Scandinavian Journal of Plastic and Reconstructive Surgery and Hand Surgery 1983;17:247–252.
[18] Garcia C, Clark RE, Buchanan M. Dermatofibrosarcoma protuberans. International Journal of Dermatology 1996;35:867.
[19] Arnaud EJ, Perrault M, Revol M, et al. Surgical treatment of dermatofibrosarcoma protuberans. Plastic and Reconstructive Surgery 1997;100:884–895.
[20] Robinson JK. Dermatofibrosarcoma protuberans resected by Mohs' surgery (chemosurgery): A 5 year prospective study. Journal of the American Academy of Dermatology 1985;12:1093–1098.
[21] Gloster HM Jr, Harris KR, Roenigk RK. A comparison between Mohs' micrographic surgery and wide surgical excision for the treatment of dermatofibrosarcoma protuberans. Journal of the American Academy of Dermatology 1996;35:82.
[22] Ratner D, Thomas CO, Johnson TM, et al. Mohs' micrographic surgery for the treatment of dermatofibrosarcoma protuberans: results of a multi-institutional series with an analysis of the extent of microscopic spread. Journal of the American Academy of Dermatology 1997;37(4):600.
[23] Picciotto F, Basolo B, Caliendo V, et al. Dermatofibrosarcoma protuberans at the site of an arteriovenous fistula in a renal transplant recipient. Transplantation 1999;68(7): 1074–1075.
[24] Brown VL, Proby CM, Harwood CA, Cerio R. Dermatofibrosarcoma protuberans in a renal transplant patient. Histopathology 2003;42(2):198–200.
[25] Lai KN, Lai F, King WWK, et al. Dermatofibrosarcoma protuberans in a renal transplant patient. Australia and New Zealead Journal of Surgery 1995;65:900–902.
[26] Coard K, Branday JM, LaGrenade L. Dermatofibrosarcoma protuberans; A 10 year clinicopathological review of an uncommon tumour. The West Indian Medical Journal 1994;43:130–133.
[27] Sherlock DJ, Rickards H, Gardecki TI, Hamer JD. Development of sarcoma in a surgical scar. Postgraduate Medical Journal 1987;63:1097.
[28] Ozyazgan I, Kontas O. Burn scar sarcoma. Burns 1999;25:455–458.
[29] Nakanishi H, Tomita Y, Yoshikawa H, Sato N, Ochi T, Aozasa K. Frequent p53 gene mutation in soft tissue sarcomas arising in burn scars. Japanese Journal of Cancer Research: Gann 1999;90:276.
[30] Weiss SW, Goldblum JR. General considerations. In Weiss SW, Goldblum JR, editors. Enzinger and Weiss's soft tissue tumours, 4th ed. St. Louis, MO: Mosby; 2001.
[31] Weedon, D. Cutaneous deposits. In Weedon, D. Skin pathology. London: Churchill Livingstone; 2002.
[32] Khan IU, Moiemen NS, Firth J, Frame JD. Malignant melanoma disguised by a tattoo. British Journal of Plastic Surgery 1999;52(7):598.
[33] Earley MJ. Basal cell carcinoma arising in tattoos: A clinical report of two cases. British Journal of Plastic Surgery 1983;36:258.
[34] McQuarry DG. Squamous cell carcinoma arising in a tattoo. Minn Med 1966;49(5):799.

